# Bone‐Targeted Delivery of Cell‐Penetrating‐RUNX2 Fusion Protein in Osteoporosis Model

**DOI:** 10.1002/advs.202301570

**Published:** 2023-08-13

**Authors:** Seoyeon Kim, Haein Lee, Jiyeon Hong, Seung Hyun L. Kim, Euntaek Kwon, Tai Hyun Park, Nathaniel S. Hwang

**Affiliations:** ^1^ School of Chemical and Biological Engineering Institute of Chemical Processes Seoul National University 1 Gwanak‐ro, Gwanak‐gu Seoul 08826 Republic of Korea; ^2^ Interdisciplinary Program in Bioengineering Seoul National University 1 Gwanak‐ro, Gwanak‐gu Seoul 08826 Republic of Korea; ^3^ BioMAX/N‐Bio Institute Institute of BioEngineerig Seoul National University 1 Gwanakro, Gwanak‐gu Seoul 08826 Republic of Korea; ^4^ Department of Nutritional Science and Food Management Ewha Womans University 52, Ewhayeodae‐gil, Seodaemun‐gu Seoul 03760 Republic of Korea

**Keywords:** bone targeting, cell‐penetrating protein, hydroxyapatite, osteogenic differentiation, osteoporosis, poly(aspartic acid)

## Abstract

The onset of osteoporosis leads to a gradual decrease in bone density due to an imbalance between bone formation and resorption. To achieve optimal drug efficacy with minimal side effects, targeted drug delivery to the bone is necessary. Previous studies have utilized peptides that bind to hydroxyapatite, a mineral component of bone, for bone‐targeted drug delivery. In this study, a hydroxyapatite binding (HAB) tag is fused to 30Kc19α‐Runt‐related transcription factor 2 (RUNX2) for bone‐targeting. This recombinant protein can penetrate the nucleus of human mesenchymal stem cells (hMSCs) and act as a master transcription factor for osteogenesis. The HAB tag increases the binding affinity of 30Kc19α‐RUNX2 to mineral deposition in mature osteoblasts and bone tissue, without affecting its osteogenic induction capability. In the osteoporosis mouse model, intravenous injection of HAB‐30Kc19α‐RUNX2 results in preferential accumulation in the femur and promotes bone formation while reducing toxicity in the spleen. These findings suggest that HAB‐30Kc19α‐RUNX2 may be a promising candidate for bone‐targeted therapy in osteoporosis.

## Graphical abstract

Bone‐targeted drug delivery for osteoporosis treatment is necessary for efficacy and safety. Herein, hydroxyapatite binding peptide (HAB) is utilized for bone‐targeted delivery of osteogenic recombinant protein (30Kc19α‐RUNX2). HAB‐30Kc19α‐RUNX2 has high binding affinity to the bone and induced osteogenesis in vitro and in vivo.

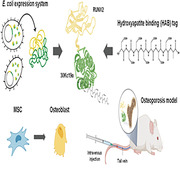

## 1 Introduction

Osteoporosis is a musculoskeletal disorder characterized by a loss of bone mass that causes fractures in hip, spine, and wrist even with the minor impact.^[^ ^1^ ^]^ The onset of osteoporosis is closely associated with aging and decreased estrogen secretion after menopause, resulting in faster bone resorption by osteoclasts compared to bone formation by osteoblasts.^[^ ^2^ ^]^ The prevalence of osteoporosis is increasing every year with the entry into an aged society and has become a serious medical and socioeconomical problem.^[^ ^3^ ^]^ Over the past few decades, extensive research has been carried out to excavate new drug candidates for the osteoporosis. Despite the dramatic progress in osteoporosis treatment, current medical treatments such as bisphosphonate, oestrogen, calcitonin, and denosumab are incomplete in terms oflong‐term stability and efficiency.^[^ ^4^ ^]^ In addition, the need for a new osteoporosis treatment is emerging due to concerns over the side effects of existing treatments.

In a previous study, as an alternative to the existing osteoporosis treatments, we developed a recombinant protein delivery system using 30Kc19α‐RUNX2, which differentiates human mesenchymal stem cells (hMSCs) into osteoblasts.^[^ ^5^ ^]^ 30Kc19α‐RUNX2 is a fusion protein of runt‐related transcription factor 2 (RUNX2), a key transcription factor for osteoblast differentiation, and 30Kc19α, a cell‐penetrating protein. RUNX2 binds to the regulatory region of osteogenic genes and activates intracellular signaling cascade related to osteoblast differentiation.^[^ ^6^ ^]^ 30Kc19α is a cell penetrable domain of 30Kc19 protein derived from silkworm hemolymph of *Bombyx mori*.^[^ ^7^ ^]^ 30Kc19α delivers cargo protein into the cells, enhances protein stability, and increases soluble protein expression in the *Escherichia coli (E. Coli)*.^[^ ^8^ ^]^ 30Kc19α with membrane penetrating function could transport RUNX2 to hMSCs across plasma membranes. In addition, due to nuclear localization sequence (NLS) within RUNX2, 30Kc19α‐RUNX2 can enter the nucleus, inducing the expression of the osteoblast‐related genes. 30Kc19α also improves the stability of the cargo protein, which results in the long, sustainable effect of the treated recombinant protein without the need for multiple repeated administration.^[^ ^5^ ^]^ At last, RUNX2 is normally formed as an inclusion body in the *E. coli* expression system, but by fusing 30Kc19α, it is expressed in a soluble form, enabling cost‐effective and mass production of recombinant proteins.

We demonstrated that 30Kc19α‐RUNX2 successfully penetrates the nucleus of hMSCs and induces bone regeneration in vitro and in vivo.^[^ ^5^ ^]^ Delivery of 30Kc19α‐RUNX2 to hMSCs increased bone mineral density, activated alkaline phosphatase (ALP) and enhanced the expression of osteogenic genes. In addition, when gelatin cryogels with 30Kc19α‐RUNX2 treated hMSCs were placed on the cranial defect in vivo, bone formation was promoted. Although 30Kc19α‐RUNX2 can be applied to osteoporosis treatment, non‐specific delivery during the systemic circulation can lead to side effects. 30Kc19α‐RUNX2 could be accumulated in the inadequate tissue, resulting in unexpected penetration of 30Kc19α‐RUNX2 into cells other than those surrounding the bone. Moreover, the therapeutic efficiency of 30Kc19α‐RUNX2 decreases in the systemic delivery because the protein distributes to other tissues and reaches less to the bone site.^[^ ^9^ ^]^ In recent studies, various hydroxyapatite, the most abundant bone mineral, binding tags have been identified for bone specific delivery.^[^ ^10^ ^]^ Those targeting moieties include oligopeptide of repeating aspartic acid (asp) or glutamic acid (glu), bisphosphonates, and tetracyclines.^[^ ^9^ ^]^ The bone binding moieties have potential benefits in that they effectively bind to hydroxyapatite and sometimes perform osteoblast activation and osteoclast inhibition.^[^ ^11^ ^]^ However, bisphosphonates can cause long‐term side effects such as atypical femur fractures, osteonecrosis of the jaw, esophageal ulcers, and atrial fibrillation.^[^ ^4c^ ^]^ Tetracyclines are known to cause permanent teeth discoloration, diarrhea, and nausea.^[^ ^12^ ^]^ Therefore, we selected oligopeptide of repeating aspartic acid as a bone binding moiety, since aspartic acid is biocompatible with high binding affinity to the hydroxyapatite.

**Figure 1:**
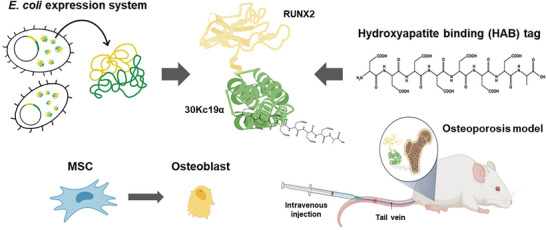
A schematic representation of the bone specific delivery of cell penetrable 30Kc19α‐RUNX2 protein for the treatment of osteoporosis. Created with BioRender.com.

Herein, we designed a bone targeting protein delivery system by conjugating hydroxyapatite binding tag (HAB; DDDDDDDC) to the 30Kc19α‐RUNX2 (**Figure** **1**). We demonstrated that HAB‐30Kc19α‐RUNX2 is soluble expressed in the *E. coli* expression system and effectively penetrates the cytoplasm and nucleus of hMSCs. To prevent 30Kc19α‐RUNX2 from penetrating cells in tissues other than bone, hydroxyapatite binding affinity was evaluated. In vitro bone mineral binding assay, *ex vivo* mouse tissue binding assay, and the live imaging of protein biodistribution confirmed that 30Kc19α‐RUNX2 effectively binds to bone minerals. Bone targeting 30Kc19α‐RUNX2 showed excellent osteogenic induction capability in vitro in hMSCs. Application of fusion protein to in vivo mouse osteoporosis model also showed that HAB‐30Kc19α‐RUNX2 enhances bone regeneration, compensating for the bone loss induced by osteoporosis. Hence, a system that delivers cell‐penetrable osteogenic protein specifically to the bone tissues would be a highly innovative and fundamental therapeutic strategy for osteoporosis treatment.

## 2 Results

### 2.1 Production of Recombinant Proteins in *E. coli* Expression System and their Low Cytotoxicity and Cell‐Penetration Ability in hMSCs

**Figure 2:**
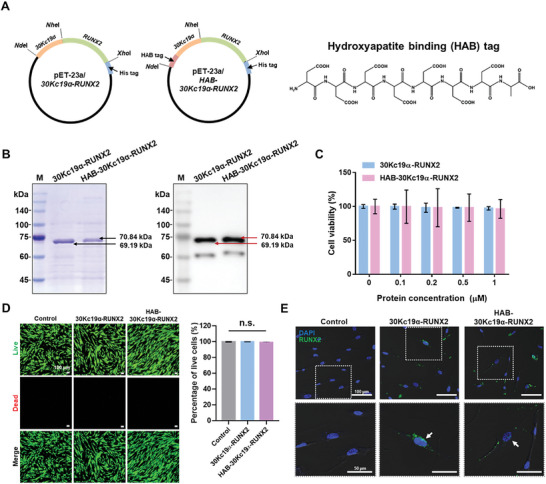
Addition of hydroxyapatite binding (HAB) tag to 30Kc19α‐RUNX2 and its effect on cytotoxicity and cell‐penetration of 30Kc19α‐RUNX2. A) Plasmid construction of 30Kc19α‐RUNX2 and HAB‐30Kc19α‐RUNX2 (left) and chemical structure of HAB (right). B) Coomassie blue staining and western blot analysis of purified recombinant proteins. Anti‐RUNX2 antibody was used as a primary antibody. M, marker. C,D) Cytotoxicity assay data showing low cytotoxicity of recombinant proteins. CELLOMAX cell viability assay data taken 72 h after treating cells with various recombinant protein concentrations (C) and Live/Dead images taken 72 h after treating cells with 1 µm of recombinant proteins (D). Quantification of the percentage of live cells is also shown in the bar graph. The percentage of dead cells was presented as fold changes relative to the control group. n.s., non‐significant. Scale bar: 100 µm. E) Confocal images of recombinant protein‐treated hMSCs showing the cell‐penetration ability of recombinant proteins. hMSCs were treated with 1 µm of the proteins for 1 h. The recombinant proteins were labelled with Alexa Fluro 488, and the nucleus with DAPI. White arrows indicate recombinant proteins located in the nucleus. Bottom images show the magnified area of the white box. Scale bar: 100 µm (up) and 50 µm (down).

*E. coli* expression vectors, pET‐23a/*30Kc19α‐RUNX2* and pET‐23a*/HAB‐30Kc19α‐RUNX2* were constructed for recombinant protein expression in *E. coli* (**Figure** **2**A). Hydroxyapatite binding (HAB) tag (DDDDDDDC) was fused to the N‐terminus of 30Kc19α‐RUNX2 for bone‐targeting delivery (Figure S1, Supporting Information). After expression and purification steps, recombinant proteins were analyzed by coomassie blue staining and western blot analysis. Figure 2B shows that both proteins were located at the theoretical sizes, of (69.19 and 70.84) kDa, respectively. hMSCs were treated with purified proteins and viability was measured using WST‐8, a highly sensitive water‐soluble tetrazolium salt. The viability was almost 100% (Figure 2C) and live and dead staining showed that there were few dead cells (Figure 2D), even when cells were exposed to 1 µm of recombinant proteins for 72 h. This indicates that both recombinant proteins have little cytotoxicity to hMSCs. Immunocytochemical analysis was conducted to evaluate the cellular membrane transduction ability of HAB‐30Kc19α‐RUNX2. Green fluorescence‐labelled proteins were in the cell cytoplasm but also in the nucleus (Figure 2E). We confirmed that HAB‐30Kc19α‐RUNX2 could penetrate cells and nuclei like 30Kc19α‐RUNX2 despite the addition of the HAB tag.

### 2.2 Hydroxyapatite Binding and Bone Affinity Properties of HAB‐30Kc19α‐RUNX2

**Figure 3:**
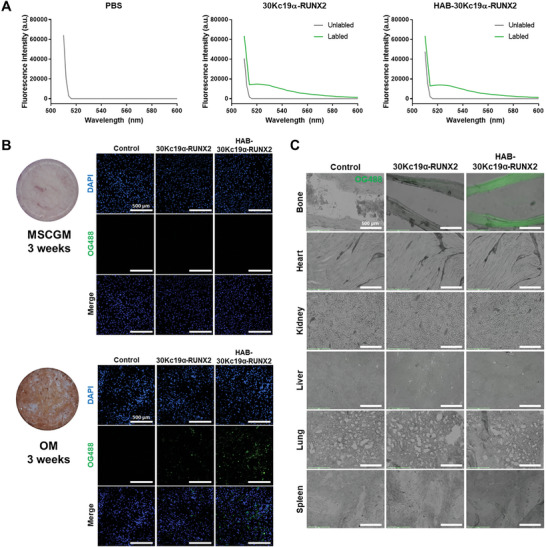
Binding affinity of HAB‐30Kc19α‐RUNX2 to osteoblast mineral deposits and bone tissue section. A) Confirmation of fluorescent dye (OG488) conjugation to recombinant proteins by measuring the fluorescence intensity of unlabel1ed and label1ed recombinant proteins. B) Comparison of binding affinities of 30Kc19α‐RUNX2 and HAB‐30Kc19α‐RUNX2 to osteoblast mineral deposits. hMSCs were cultured with MSCGM or OM for 3 weeks. Then, cells were fixed with 4% PFA and treated with 1 µm of OG488‐label1ed recombinant proteins. Scale bar: 500 µm. C) Comparison of binding affinities of recombinant proteins to a bone tissue section. Mouse tissue sections were treated with 1 µm of OG488‐label1ed recombinant proteins. Bright‐field and green, fluorescent images were merged using ImageJ software. Scale bar: 500 µm.

Poly(aspartic acid) has been exploited for bone targeting of various forms of drugs (peptides, nanoparticles, liposomes) in that it has a high affinity with hydroxyapatite, the main component of the bone.^[^ ^13^ ^]^ This affinity is the result of the calcium chelation of poly(aspartic acid) and the complex formation with calcium.^[^ ^14^ ^]^ Therefore, poly(aspartic acid) was chosen as a HAB tag to impart hydroxyapatite binding and bone targeting abilities to the fusion protein. In this section, the hydroxyapatite binding ability of HAB‐tagged protein was confirmed by incubating proteins with hydroxyapatite‐containing materials. Before binding assays, the C‐terminus of the recombinant proteins was conjugated with a green, fluorescent dye, Oregon Green 488 (OG488). Then, fluorescence intensities of unlabelled and labelled proteins were measured in a microplate reader. As shown in **Figure** **3**A, the fluorescence intensity of the labelled protein increased at 526 nm (λ_em_ of OG488) than that of the unlabelled protein, confirming OG488 conjugation to the recombinant proteins. To evaluate the binding affinities of HAB‐30Kc19α‐RUNX2 to bone minerals, osteogenic differentiation was induced by changing hMSCs medium into an osteogenic medium (OM) (Figure 3B). As a control group, hMSCs were cultured in the mesenchymal stem cell growth medium (MSCGM). After 3 weeks of treatment, Alizarin Red S (ARS) staining was conducted to both MSCGM‐cultured hMSCs and OM‐cultured hMSCs. OM‐cultured hMSCs showed stronger red staining than the MSCGM‐cultured hMSCs, suggesting that a higher level of calcium bone minerals was formed in the OM‐cultured hMSCs. In the early stage of bone mineralization, calcium is constantly converted into hydroxyapatite due to chemical‐physical transformations performed by the cellular activity of hMSCs, so hydroxyapatite is the most commonly formed mineral among bone minerals.^[^ ^15^ ^]^ After confirming matrix mineralization in the OM‐cultured hMSCs, the medium was removed and PBS, OG488‐labelled recombinant proteins were applied to both differentiated and undifferentiated cells. DAPI was counterstained to show that the number of cells in each group was similar. OG488‐labelled HAB‐30Kc19α‐RUNX2 showed the highest green fluorescence intensity in OM‐cultured hMSCs, indicating that HAB‐30Kc19α‐RUNX2 has a strong binding affinity to bone minerals, while PBS and OG488‐labelled 30Kc19α‐RUNX2 showed less or no green fluorescence in OM‐cultured hMSCs indicating little binding affinity to bone minerals. A significant difference in green fluorescence intensity between those groups suggests that the presence of HAB tag determines the binding force of protein to the bone matrix. In contrast, both OG488‐labelled 30Kc19α‐RUNX2 and HAB‐30Kc19α‐RUNX2 did not bind to MSCGM‐cultured hMSCs, showing that proteins, even HAB‐tagged proteins, could not bind to the cell culture environment with low mineral density. After confirming that HAB‐30Kc19α‐RUNX2 has a binding affinity to bone minerals in the cell culture plate, we evaluated whether the protein has a binding affinity to bone tissues (Figure 3C). For ex vivo mouse tissue binding assay, tissues including femur, heart, kidney, liver, lung, and spleen were collected, sectioned, and treated with fluorescence‐labelled recombinant proteins. Interestingly, OG488‐labelled HAB‐30Kc19α‐RUNX2 is bound only to the femur but not to other tissues. In contrast, OG488‐labelled 30Kc19α‐RUNX2 did not bind to tissues including the femur, heart, kidney, liver, lung, and spleen. This means that HAB‐30Kc19α‐RUNX2 could target only bone tissues, thereby minimizing unwanted consequences induced by non‐specific binding of recombinant proteins to other tissues.

### 2.3 In Vitro Osteogenic Induction Capability of HAB‐30Kc19α‐RUNX2

**Figure 4:**
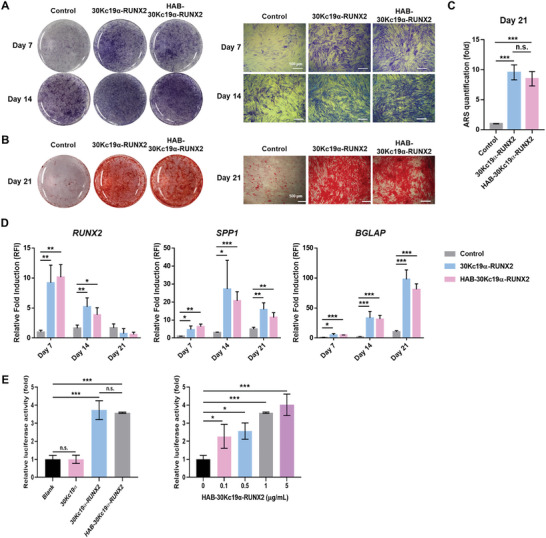
In vitro osteogenic differentiation of HAB‐30Kc19α‐RUNX2‐treated hMSCs. A–C) ALP staining and ARS staining data showing ALP expression and calcium deposition in hMSCs. hMSCs were treated with 200 nm of 30Kc19α‐RUNX2 or HAB‐30Kc19α‐RUNX2. ALP staining was performed on hMSCs cultured for (7 and 14) days in OM (A). ARS staining was performed on hMSCs cultured for 21 days in OM (B). ARS quantification is shown in bar graph (C). Scale bar: 500 µm. ***p < 0.001, n.s., non‐significant. D) Assessment of osteogenic marker gene expression profile (*RUNX2*, *SPP1*, and *BGLAP*) via RT‐qPCR. hMSCs cultured for 7, 14, 21 days in OM were examined. mRNA expression levels of osteogenic marker genes were normalized to *GAPDH*. Then, the normalized values were expressed as relative fold induction (RFI) over the control group on day 7. *p < 0.05, **p < 0.01, ***p < 0.001.

A previous study confirmed the osteogenic induction capability of 30Kc19α‐RUNX2.^[^ ^5^ ^]^ To figure out whether HAB tag does not impair the osteogenic properties of 30Kc19α‐RUNX2, hMSCs were treated with either 30Kc19α‐RUNX2 without or with HAB tag. Then, the ability to induce osteogenic differentiation of hMSCs was analyzed by staining and real‐time qRT‐PCR. First, hMSCs were stained with ALP detecting dye, since ALP is expressed in the early stage of osteogenesis.^[^ ^16^ ^]^ ALP can be detected using Naphthol AS‐MX phosphate as a substrate, which stains ALP‐expressing cells purple. On day 7, both recombinant protein‐treated groups displayed intense purple color, whereas the control group showed little purple color (**Figure** **4**A). On day 14, the purple color was getting more intense in all three groups. No significant difference was observed in the degree of purple staining between 30Kc19α‐RUNX2 and HAB‐30Kc19α‐RUNX2‐treated group. Next, late osteogenic differentiation of hMSCs was confirmed by ARS staining. Mineral deposits are an indication of mature osteoblast and are stained red by ARS. Figure 4B shows that 30Kc19α‐RUNX2‐ and HAB‐30Kc19α‐RUNX2‐treated group accumulated more mineral deposits than the control group on day 21. According to ARS quantification, mineralization was increased by 9.57‐ and 8.50‐fold, respectively in the 30Kc19α‐RUNX2 or HAB‐30Kc19α‐RUNX2 group compared to the control group (Figure 4C). As with the ALP staining results, there was no statistically significant difference between the 30Kc19α‐RUNX2‐treated group and the HAB‐30Kc19α‐RUNX2 treated group. Finally, osteoblast‐related gene expression was quantified via real‐time qRT‐PCR analysis. Three osteoblast‐specific genes expressed in pre‐osteoblast (*RUNX2*) and mature osteoblast (*secreted phosphoprotein 1* (*SPP1*; gene of osteopontin), and *bone gamma‐carboxyglutamate protein* (*BGLAP*; gene of osteocalcin)) were selected. Real‐time qRT‐PCR data analysis showed that both 30Kc19α‐RUNX2‐ and HAB‐30Kc19α‐RUNX2‐treated group exhibited higher expression of *RUNX2, SPP1*, and *BGLAP* compared to control group (Figure 4D). Only on day 21, the expression level of *RUNX2* in the experimental group was lower than that of the control group. As *RUNX2* is known as an early osteogenic marker, it seems that *RUNX2* was downregulated in the late osteogenic differentiation. Again, no statistical significance was found between the two experimental groups. The role of 30Kc19α‐RUNX2 and HAB‐30Kc19α‐RUNX2 as transcription factors were also investigated using a 2472 bp BSP promoter reporter plasmid containing the native BSP promoter fragment, provided by Renny Franceschi (University of Michigan Medical School) (Figure 4E). The promoter of this plasmid contains two putative RUNX2‐binding sites having the consensus sequence RC‐CRC(A/T) and the luciferase reporter in the plasmid. This BSP‐luciferase reporter construct examined the involvement of 30Kc19α‐RUNX2 and HAB‐30Kc19α‐RUNX2 in BSP expression. When NIH3T3 cells transfected with BSP promoter reporter plasmid were treated with recombinant proteins for 4 h, luciferase expression was upregulated compared to the blank or 30Kc19α treated groups. In addition, we evaluated the level of luciferase expression as the concentration of HAB‐30Kc19α‐RUNX2 increased. We treated HAB‐30Kc19α‐RUNX2 with different concentration (0, 0.1, 0.5, 1, 5 µg mL^−1^) and confirmed that the level of luciferase expression increased as the concentration of protein increased.

Taken together, it was confirmed that the HAB tag enhanced hydroxyapatite binding and bone affinity of 30Kc19α‐RUNX2 without any effect on the osteogenic induction properties of it.

### 2.4 Biodistribution of HAB‐30Kc19α‐RUNX2

**Figure 5:**
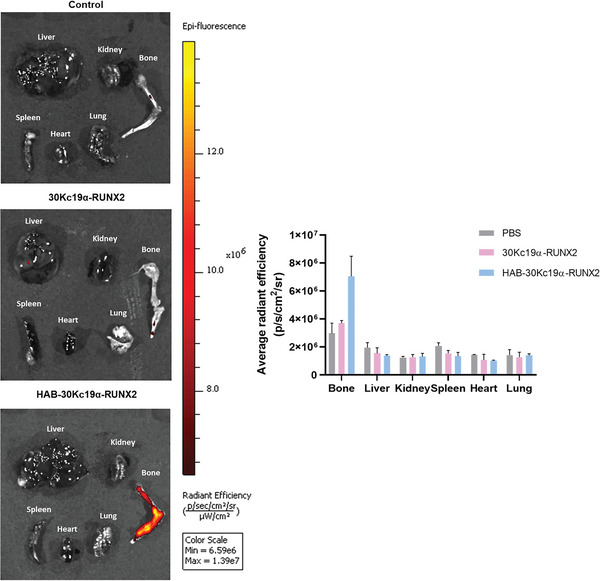
Biodistribution of HAB‐30Kc19α‐RUNX2. IVIS in vivo imaging was taken to track recombinant proteins within the body. The bone‐specific delivery of HAB‐30Kc19α‐RUNX2 and the localization of 30Kc19α‐RUNX2 were assessed. Balb/c‐nu mice were administered with Atto550‐label1ed recombinant proteins (50 µg, 0.25 µg µL^−1^) in PBS intravenously (*n* = 3). After 2 h, major organs were collected for fluorescence imaging (554/576 nm Excitation/Emission). For accurate comparison between different groups, the minimum and maximum values of all groups were unified (6.59e6/1.39e7 Minimum/Maximum). The average radiant efficiency of the region of interest (ROI) was measured after imaging.

To investigate the systemic distribution of recombinant proteins in vivo, NTA‐Atto550 was labelled 30Kc19α‐RUNX2 and HAB‐30Kc19α‐RUNX2. Atto550‐labelled 30Kc19α‐RUNX2 and HAB‐30Kc19α‐RUNX2 were injected to the mice via tail vein injection, and after 2 h mice were sacrificed and major organs (liver, heart, bone of femur and tibia, spleen, kidney, and lung) were collected for IVIS fluorescence imaging. **Figure** **5** shows that fluorescence from HAB‐30Kc19α‐RUNX2 was detected mostly in bones, and some in the liver, indicating that injected HAB‐30Kc19α‐RUNX2 circulates along the blood vessel and then moves specifically to the bone tissue by the binding force of HAB and hydroxyapatite. The fluorescence signal of Atto550‐labelled HAB‐30Kc19α‐RUNX2 group had the strongest fluorescence signal in the bone tissue compared to the other groups. On the contrary, Atto550‐labelled 30Kc19α‐RUNX2 group showed a weak fluorescence signal in the bone, which was consistent with the results of an ex vivo mouse tissue binding assay (Figure 3C). In Atto550‐labelled 30Kc19α‐RUNX2 group, protein accumulated mainly in the liver and neither HAB‐30Kc19α‐RUNX2 group nor 30Kc19α‐RUNX2 group revealed fluorescence in the kidney, spleen, heart, and lung. Since the protein biodistribution was confirmed only 2 h after intravenous injection of the proteins, there is a possibility that recombinant proteins could accumulate in other organs in the long term.

### 2.5 In Vivo Osteogenic Induction Capability of HAB‐30Kc19α‐RUNX2

**Figure 6:**
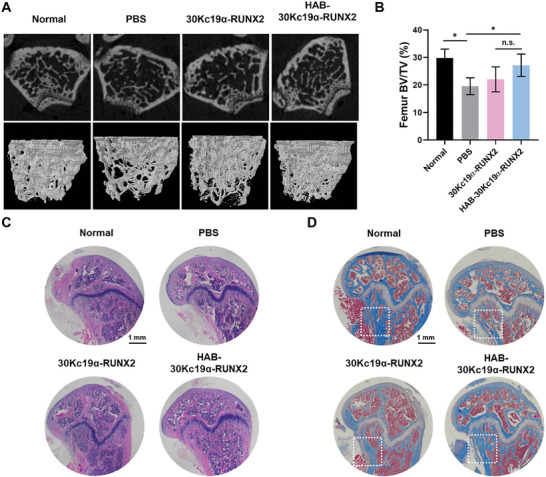
In vivo bone regeneration of HAB‐30Kc19α‐RUNX2 treatment in postmenopausal osteoporosis model. A,B) Micro‐CT analysis for new bone formation after recombinant protein treatments. Four weeks after the osteoporosis model was generated by ovariectomy (OVX) in Balb/c mice, PBS (50 µL), 30Kc19α‐RUNX2 (50 µg, 1 µg µL^−1^) and HAB‐30Kc19α‐RUNX2 (50 µg, 1 µg µL^−1^) were injected via tail vein injection four times every 1 week (*n* = 3). The micro‐CT image of the distal femur was reconstructed into a 3D image, showing the alternation in trabecular bone density (A). Bone volume to total volume fraction (BV/TV) was also measured (B). *p < 0.05. C) H&E staining showing the histomorphological change in the femur. Scale bar: 1 mm. D) MTC staining showing collagen and mineralized bone formation. Scale bar: 1 mm.

In a previous study, it was demonstrated that 30Kc19α‐RUNX2 penetrated the nucleus and promoted new bone formation.^[^ ^5^ ^]^ HAB‐30Kc19α‐RUNX2 protein delivery system developed further from the 30Kc19α‐RUNX2 protein system, allowing bone‐specific delivery by conjugating HAB tag that has a specific binding affinity to hydroxyapatite in bones (Figure 3 and Figure 5). To investigate whether the bone targeting system of HAB‐30Kc19α‐RUNX2 is effective in new bone formation, an animal experiment was conducted using the postmenopausal osteoporosis model. During menopause, the normal bone remodeling process is disrupted by the failure of the ovary in estrogen secretion. Normally, estrogen adheres to the osteoclast receptor and inhibits the activation and formation of osteoclast.^[^ ^17^ ^]^ In addition, recent studies have also found that estrogen promotes the differentiation of osteoblast and inhibits apoptosis.^[^ ^18^ ^]^ When estrogen hormone rapidly decreases after menopause, those estrogen‐induced effects diminish, accelerating the rate of bone resorption than that of the formation.^[^ ^17^ ^]^ Applying this physiological phenomenon, the osteoporosis model was established by ovariectomy (OVX). After 4 weeks of ovarian removal, PBS, equal amount of 30Kc19α‐RUNX2, and HAB‐30Kc19α‐RUNX2 were intravenously injected once a week for a total of 4 weeks. After that, mice were sacrificed, and femur specimens were collected for micro‐CT analysis. A femur was chosen among various bone sites for osteoformation analysis because long bones such as the femur are more sensitive to OVX than other bone tissues such as spine and cranial bone.^[^ ^19^ ^]^ The micro‐CT image was reconstructed into a 3D image and bone volume to total volume fraction (BV/TV) was measured using CT_An_ software. As shown in **Figure** **6**A,B, the group administered PBS after OVX had lower bone density and BV/TV ratio than the normal group without OVX. This data shows that the OVX‐induced osteoporosis model was well constructed, which was also proven by the reduction in uterus size and weight in OVX groups compared to normal groups (Figure S2, Supporting Information). In addition, micro‐CT images of femur revealed that the HAB‐30Kc19α‐RUNX2 group formed the greatest amount of bone among OVX groups with the highest BV/TV ratio. The BV/TV ratio of HAB‐30Kc19α‐RUNX2 was 29.08% on average while PBS and 30Kc19α‐RUNX2 were 19.54% and 22.04%, respectively. The BV/TV ratio of the normal group was 29.76%, suggesting that bone loss of HAB‐30Kc19α‐RUNX2 recovered to a degree like the trabecular density of normal bone. This result appears to be due to the bone‐specific delivery of recombinant proteins by HAB tag. Although the HAB‐30Kc19α‐RUNX2 group exhibited similar or slightly less osteogenic capabilities than the 30Kc19α‐RUNX2 group in in vitro osteogenic differentiation assays (Figure 4A,B; Figure S3, Supporting Information), the HAB‐30Kc19α‐RUNX2 group promoted bone formation similar to or higher than the 30Kc19α‐RUNX2 group in animal experiments. This shows that even though HAB‐30Kc19α‐RUNX2 has similar or less ability to differentiate hMSCS into osteoblasts compared to 30Kc19α‐RUNX2, it has a better targeting ability to generate bone in vivo. The 3D micro‐CT images and BV/TV values demonstrate that bone‐specific delivery of recombinant osteogenic proteins is effective in treating osteoporosis.

**Figure 7:**
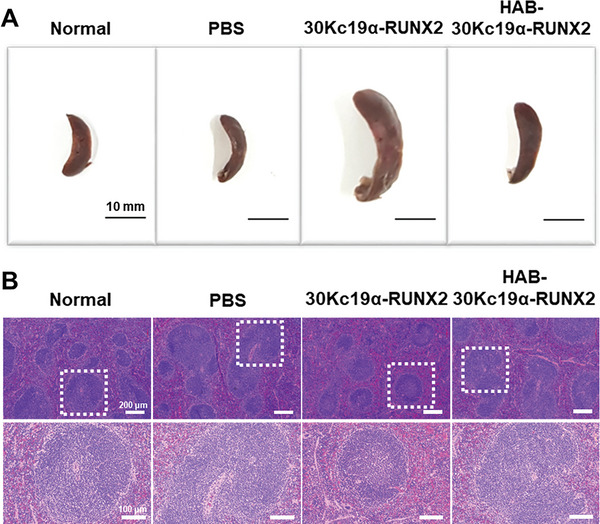
Morphological and cytological changes in the spleen after the treatment of recombinant proteins. A) Splenomegaly observed after the treatment of 30Kc19α‐RUNX2 and HAB‐30Kc19α‐RUNX2. Following 4 weeks of protein injection, spleens were dissected to evaluate protein delivery to the spleen and the immune response induced by external protein injection. Scale bar: 10 mm B) H&E staining showing the cellular and histomorphological change in the spleen.

To analyze histomorphological changes in distal femur, hematoxylin & eosin (H&E) and Masson's trichrome staining (MTC) were performed (Figure 6C,D). In the H&E staining, cell nucleus is shown dark blue while the cytoplasm is shown pink. After 4 weeks of protein treatment, the number of trabecular cells in the distal femur decreased in the PBS group, while in the rest of the group, the number of cells recovered normal. This once again shows that osteoporosis was successfully generated in OVX group, and the treatment of recombinant proteins can compensate for bone loss induced by osteoporosis. In addition, 30Kc19α‐RUNX2 and HAB‐30Kc19α‐RUNX2 groups were stained pink more than the PBS group, which indicates an increase in femur extracellular matrix density. The result of MTC staining was also consistent with H&E staining. In the MTC staining, collagen is stained blue while cytoplasm or muscle is stained red. There was a significant difference in blue staining between PBS and recombinant protein groups in that 30Kc19α‐RUNX2 and HAB‐30Kc19α‐RUNX2 were stained blue on a larger area than the PBS group. This observation indicates that more collagen was produced in 30Kc19α‐RUNX2 and HAB‐30Kc19α‐RUNX2 group compared to the PBS group, which means that OVX‐induced osteoporosis was recovered by new bone formation in the trabecular region. Micro‐CT and histological analysis demonstrate the excellent bone‐forming ability of the HAB‐30Kc19α‐RUNX2 due to a system that can effectively deliver protein to bones. In **Figure** **7**A, splenomegaly was observed in the 30Kc19α‐RUNX2 group as an immune response to external protein injection. On the contrary, the spleen of the HAB‐30Kc19α‐RUNX2 group, even though it was slightly larger than the normal or PBS group, displayed less spleen enlargement compared to the 30Kc19α‐RUNX2 group, indicating that HAB‐30Kc19α‐RUNX2 moved more specifically to the bone and less to the spleen. Although recombinant protein evokes weak immune responses, no difference was observed between groups in the H&E of spleens (Figure 7B).

## 3 Discussion

RUNX2 functions as a master regulator of osteogenic differentiation. RUNX2 associates with the promoter region or the RUNX2‐responsive region of the osteogenic marker genes and commits the differentiation of human mesenchymal stem cells (hMSC) into osteoblasts.^[^ ^20^ ^]^ Osteogenic marker genes such as *collagen type I (COL1A1), osteocalcin (OCN), alkaline phosphatase (ALP), osteopontin (OPN)*, and *bone sialoprotein (BSP)* are upregulated by the activity of RUNX2.^[^ ^21^ ^]^ Several research reports validated that even a single delivery of RUNX2 to hMSC induces direct cellular conversion into osteoblasts, leading to bone formation.^[^ ^22^ ^]^ The delivery of RUNX2 to hMSCs for transdifferentiation into osteoblasts can be a potential strategy to treat osteoporosis.

However, the challenges of delivering RUNX2 to the hMSCs still remain due to safety issues. Previously, viral vectors such as adenoviral and retroviral vectors were widely used for the delivery of genes.^[^ ^23^ ^]^ These gene delivery systems pose one of the greatest problems by getting permanently integrated into an undesired location of the genome and causing oncogenic mutation.^[^ ^24^ ^]^ Another gene delivery method, non‐viral vectors using cationic polymers or lipids, show low transfection efficiency and have the limitation in the transgene size.^[^ ^25^ ^]^ Of the genes treated to cells, 36% can reach the cytosol, but only 0.01% can reach the nucleus and half get a chance to be transcribed.^[^ ^26^ ^]^ Protein‐based RUNX2 delivery system can overcome these limitations of the existing delivery systems. Herein, recombinant RUNX2 protein was fused with 30Kc19α for its effective delivery into the cells. 30Kc19α is a cell‐penetrating protein that could deliver cargo proteins directly into cytosols by endocytosis or direct penetration.^[^ ^27^ ^]^ Unlike gene delivery systems, transcription and translation processes are not required in 30Kc19α‐mediated protein delivery system. 30Kc19α also could improve the stability of the cargo protein, resulting in the treated recombinant protein's prolonged, sustained effect.^[^ ^28^ ^]^ Therefore, 30Kc19α could deliver RUNX2 with no risk of gene insertional mutagenesis and tumor formation and pass through the hMSC membrane with low cytotoxicity and high efficiency.^[^ ^5^ ^]^

Although 30Kc19α‐RUNX2 is an effective delivery system for osteogenesis, non‐specific binding to organs other than bones can cause side effects and reduce drug efficacy. Therefore, aspartic acid (Asp), having a strong binding affinity to bone hydroxyapatite, was used.^[^ ^9^ ^]^ Hydroxyapatite tag (HAB), was fused to the N‐terminus of 30Kc19α‐RUNX2 protein for targeted bone delivery. Aspartic acid contains several carboxylic acids that prefer hydroxyapatite crystals that are abundantly exposed in bone turnover sites.^[^ ^29^ ^]^ Also, as aspartic acid is naturally synthesized in our body, HAB is safe and biocompatible. In this study, HAB‐30Kc19α‐RUNX2 showed no cytotoxicity in the WST‐8 viability assay and Live/Dead staining assay.

To evaluate the binding ability of HAB‐30Kc19α‐RUNX2 to bone minerals, recombinant protein was labelled with OG488 or Atto550 fluorescent dye. OG488‐labelled HAB‐30Kc19α‐RUNX2 effectively adsorbed bone mineral rich cell culture substrates. In addition, fluorescence intensities in the bone tissues were significantly higher in OG488‐labelled HAB‐30Kc19α‐RUNX2 group as compared to OG488‐labelled 30Kc19α‐RUNX2 group or control group. The in vivo biodistribution of Atto550‐labelled HAB‐30Kc19α‐RUNX2 also showed that HAB‐30Kc19α‐RUNX2 preferentially binds to bone tissues after intravenous injection. This demonstrates that HAB serves as an effective bone binding moiety to deliver recombinant proteins to the bone site.

HAB‐30Kc19α‐RUNX2 had osteogenic effect in vitro and in vivo. In the in vitro study, hMSCs showed osteoblast‐like properties after the treatment of HAB‐30Kc19α‐RUNX2. Since RUNX2 is an early transcription factor mediating osteogenesis, recombinant protein was treated in the early stage of hMSC differentiation.^[^ ^20^ ^]^ HAB‐30Kc19α‐RUNX2 treatment upregulated the expression of alkaline phosphatase and bone mineral deposits. This implies that NLS within RUNX2 mediates transport of HAB‐30Kc19α‐RUNX2 into the cell nucleus and this activates RUNX2 downstream signaling pathways associated with osteogenic marker genes. Furthermore, osteogenic induction capability of HAB‐30Kc19α‐RUNX2 revealed that fusion with 30Kc19α does not affect the inherent functionality of RUNX2.

In vivo osteoporosis model also demonstrated the osteogenic induction capability of HAB‐30Kc19α‐RUNX2. Before the experiment, postmenopausal osteoporosis model was established by removing both ovaries in the mice. Ovariectomy significantly reduces the amount of estrogen secreted from the ovary. The main action of estrogen in bone remodeling process is to inhibit bone resorption. Estrogen adheres to the osteoclast receptor and activates osteoclast apoptosis. Estrogen also suppresses osteoclastogenesis signaling pathways to prevent osteoclast formation and RANKL‐induced differentiation.^[^ ^17^ ^]^ Recent studies have demonstrated that estrogen acts in bone formation by promoting osteoblast formation and differentiation, thereby inducing bone formation.^[^ ^18^ ^]^ When estrogen rapidly decreases after ovariectomy, estrogen‐induced effects diminish, resulting in the imbalance between bone formation and resorption during bone turnover.^[^ ^17^ ^]^ The rate of bone resorption accelerates the rate of bone formation, which leads to osteoporosis. After the generation of osteoporosis model, mice were treated with HAB‐30Kc19α‐RUNX2 via tail vein injection. Micro‐CT analysis of femur revealed that the HAB‐30Kc19α‐RUNX2 group formed the greatest amount of bone among OVX groups (PBS, 30Kc19α‐RUNX2) with the highest BV/TV ratio. Also, histological analysis showed that the HAB‐30Kc19α‐RUNX2 group had the highest collagen matrix with the highest trabecular cell number. However, due to the small sample size of only three mice per group in the experiment, the statistical power to detect small significant differences between the groups may have been insufficient.^[^ ^30^ ^]^ Despite the limitation in sample size, These results indicate that the HAB‐30Kc19α‐RUNX2 delivery system marks a synergistic effect in osteogenesis by combining intrinsic osteogenic effect of RUNX2 and bone targeting effect of HAB. This targeting system is safe and reliable without the risk of genetic modification, oncogenesis, and random distribution of recombinant protein in the body.

## 4 Conclusion

Drug delivery system for bone targeting may lower the risk of indiscriminate drug distribution in the body and enhance drug efficacy by increasing drug concentration at the bone tissue. Herein, we successfully developed a therapeutic agent to treat osteoporosis, consisting of polyaspartic acid moieties (HAB) serving as a bone‐targeting ligand and 30Kc19α‐RUNX2 fusion protein as a cell‐penetrable bone‐forming protein complex. HAB‐30Kc19α‐RUNX2 penetrated the nucleus of hMSCs and induced osteogenic differentiation. In addition, HAB promoted targeted delivery of 30Kc19α‐RUNX2 to the bone mineral (hydroxyapatite)‐rich sites, allowing protein accumulation to an effective concentration at the bone tissue. This consequently promoted bone formation in an in vivo osteoporosis model. These results indicate that HAB‐30Kc19α‐RUNX2 may be a promising bone‐targeting drug to treat osteoporosis.

## 5 Experimental Section

### Plasmid Construction

pET‐23a/*30Kc19α‐RUNX2* was constructed as described in a previous study.^[^ ^5^ ^]^ Briefly, *30Kc19α* and *RUNX2* were inserted into (*Nde*I and *Nhe*I) and (*Nhe*I and *Xho*I), respectively. HAB tag sequence (GATGATGATGATGATGATGATTGC) containing forward primer was used in a polymerase chain reaction (PCR) for the insertion of HAB tag at the 5‘‐end of the *30Kc19α* gene. PCR products were purified with HiYield Gel/PCR DNA mini kit (Real Biotech Corporation (RBC), Taiwan), and then inserted into *E. coli* expression vector, pET‐23a with His tag at C‐terminus (Novagen, USA).

### Protein Expression and Purification

30Kc19α‐RUNX2 and HAB‐30Kc19α‐RUNX2 proteins were produced as described in the previous study.^[^ ^5^ ^]^ pET‐23a/*30Kc19α‐RUNX2* and pET‐23a/*HAB‐30Kc19α‐RUNX2* plasmid were transformed into the *E. coli* strain BL21 (DE3; Novagen) for protein production. The transformed *E. coli* was incubated in Luria‐Bertani broth (LB; Miller, USA) with 100 µg mL^−1^ of ampicillin (Sigma, USA) at 37 °C with the agitation speed of 200 rpm. The inoculated medium was transferred to 1 L LB broth medium and cultured at 37 °C with agitation speed of 180 rpm. When cell density reached OD_600_ of 0.4‐0.6, 1 mM of isopropyl‐β‐D‐thiogalactopyranoside (IPTG; Calbiochem, USA) was added for protein induction, and cells were further cultured for 6 h at 25 °C with the agitation speed of 180 rpm. The cells were harvested by centrifuging at 7 000 rpm for 10 min at 4 °C. Then the cell pellets were resuspended with His‐binding buffer {20 mM imidazole (Sigma), 20 mM Tris‐HCl (Sigma), and 0.5 M NaCl (Junsei, Japan), pH 8.0} and lysed with ultra‐sonication. Sonication was performed using Q500 sonicator (Q500 Sonicator, Qsonica) using the following settings: samples in ice bath, 20% amplification for 10 min and 5 sec on/5 sec off pulses. The cell lysates were centrifuged at 12 000 rpm for 30 min at 4 °C to collect soluble protein remaining in the supernatant. The supernatants were filtered using 0.2 µm bottle top filter (Jetbiofil, South Korea) before purification and were loaded into His‐Trap HP column (Cytiva, USA) prefilled with His‐binding buffer, and 0.5 M NaCl (Junsei, Japan), pH 8.0}. 30Kc19α‐RUNX2 and HAB‐30Kc19α‐RUNX2 protein were purified using nickel affinity chromatography with AKTA purification system (Cytiva). His‐washing buffer {50 mM imidazole, 20 mM Tris‐HCl, and 0.5 M NaCl, pH 8.0} was loaded on the column to remove unbound proteins, and then His‐elution buffer {350 mM imidazole, 20 mM Tris‐HCl, and 0.5 M NaCl, pH 8.0} was loaded to elute bound proteins. After the purification, buffer exchange was carried out to exchange protein solution into phosphate‐buffered saline (PBS; Sigma) using HiTrap desalting column (Cytiva) for in vitro and in vivo experiments. Finally, the diluted proteins were poured into Amicon® ultra‐15 centrifugal filters (10 kD cutoff filter; Merck, USA) and concentrated in a swing bucket centrifuge (Eppendorf) at 4000 rpm and 4 °C until the desired concentration was reached. After dilution, endotoxin determination was performed with Pierce Chromogenic Endotoxin Quant Kit (Thermo Fisher Scientific, USA) according to the manufacturer instructions. 30Kc19α‐RUNX2 and HAB‐30Kc19α‐RUNX2 both contained 0.86 endotoxin EU/mL.

### Coomassie Blue Staining and Western Blot Analysis

Sodium dodecyl sulfate‐polyacrylamide gel electrophoresis (SDS‐PAGE) was performed to identify recombinant 30Kc19α‐RUNX2 and HAB‐30Kc19α‐RUNX2. Purified proteins were mixed with 2× Laemmli sample buffer (Bio‐Rad, USA), and loaded to 10% SDS‐PAGE gel. For Coomassie blue staining, gels were stained with Coomassie Blue R‐250 (Sigma). For western blot analysis, the proteins on the gel were transferred to the nitrocellulose membrane (Cytiva). Anti‐RUNX2 antibody (Santa Cruz Biotechnology, USA) was used as a primary antibody, and goat anti‐mouse IgG‐HRP antibody (AbFrontier, Korea) was used as a secondary antibody. The membrane was treated with TOPview™ ECL pico plus western substrate (Enzynomics, Korea) to detect the protein of interest, and images were obtained by G:BOX Chemi XRQ (Syngene, UK).

### Cytotoxicity Assay

The viabilities of hMSCs exposed to different concentrations (0 to 1 µm) of recombinant proteins for 72 h were determined by CELLOMAX cell viability assay kit (Precaregene, Korea). hMSCs were seeded at 5000 cells cm^−2^ with MSCGM (Lonza, Switzerland) prior to the experiments. The next day, cells were exposed to recombinant proteins for 72 h. After that, 10% v/v of CELLOMAX cell viability assay kit solution was added to each well of the plate, and the plate was further incubated for 2 h. Absorbance at 450 nm was measured with a microplate reader (SPARK 10 M; Tecan, Switzerland). Cytotoxicity of the purified proteins was also evaluated by the LIVE/DEAD™ viability/cytotoxicity kit (Invitrogen, USA). After treating recombinant protein (1 µm) to the hMSCs for 72 h, staining was conducted following the manufacturer's instruction. The calcein AM/ethidium homodimer‐stained samples were observed via Eclipsed Ti2 inverted microscopy (Nikon, Japan) and the image was analyzed by NIS‐Elements AR software‐Nikon Instruments Inc.

### Cell‐Penetration Assay

Cell penetration ability of recombinant proteins was observed with immunocytochemical analysis. hMSCs were seeded into the confocal dish (SPL Life Science, Korea) with MSCGM. When confluency reached 50%, cells were treated with recombinant proteins (1 µm) for 4 h. Following the fixation and permeation steps, proteins were labelled with primary antibodies. Anti‐RUNX2 antibody was used as the primary antibody. Subsequently, Alexa Fluor 488 (Invitrogen) was used as a secondary antibody for the anti‐RUNX2 antibody. Nuclei were counterstained with 4′,6‐diamidino‐2‐phenylindole (DAPI; Invitrogen). Confocal laser scanning microscopy (LSM710; Carl Zeiss, Germany) was used to obtain images of cell cross‐sections. Fluorescence images were merged using Image J software (NIH, USA).

### hMSC Culture and Differentiation

hMSCs derived from bone marrow (Lonza) were cultured in a 24‐well plate (Eppendorf, Germany) with MSCGM (Lonza). When the cells reached 70% confluency, the medium was changed to OM containing recombinant proteins (200 nm). OM was prepared according to the composition described in the following reference.^[^ ^31^ ^]^ The medium was replaced with fresh OM every 2–3 days for (7, 14, and 21) days.

### Fluorescent Dye Conjugation

Fluorescent dye‐labelled recombinant proteins were prepared by mixing equal volumes of HIS Lite™ OG488‐Tris NTA‐Ni complex (AAT Bioquest, USA) and recombinant proteins in PBS. For another fluorescent dye‐labelled recombinant protein, equal volumes of NTA‐Atto550 (Merck, USA) and recombinant proteins were mixed in PBS. Those mixtures were incubated at 4 °C overnight, then free dyes were eliminated by size exclusion chromatography.

### In Vitro Cell Culture Binding Assay

When hMSCs cultured in MSCGM reached 70% confluency, cells were divided into two groups, one cultured in MSCGM and the other cultured in an OM. After 3 weeks of cell culture, ARS staining was conducted to evaluate the mineral deposits of hMSCs. Cells were fixed with 4% paraformaldehyde (PFA; Sigma) for 10 min and then incubated with PBS, OG488‐labelled recombinant proteins (1 µm) in PBS for 1 h at room temperature. After that, cell culture plates were rinsed three times with PBS to wash any unbound proteins and were counterstained with DAPI. Binding affinities of OG488‐labelled 30Kc19α‐RUNX2 and OG488‐labelled HAB‐30Kc19α‐RUNX2 to cell cultures were visualized by Eclipsed Ti2 inverted microscopy. In the microscope, a blue fluorescence filter and green fluorescence filter were used, and the two images were merged using Image J software.

### Ex Vivo Mouse Tissue Binding Assay

*Ex vivo* mouse tissue binding assay was performed as described in the previous paper.^[^ ^32^ ^]^ Balb/c‐nu mice purchased from Orient Bio (Korea) were sacrificed with carbon dioxide before mouse dissection (*n* = 3). Major organs and tissues including the femur, heart, kidney, liver, lung, and spleen were harvested and placed in PFA at 4 °C for 3 days to preserve tissue morphology. All organs and femurs were transferred to 15% sucrose (Sigma) in PBS overnight at 4 °C, and then to 30% sucrose in PBS overnight at 4 °C. The organs and femur were then removed from the sucrose and placed in an optimal cutting temperature compound (OCT; Sakura, USA). OCT embedded tissue blocks were frozen completely using dry ice and liquid nitrogen and then stored in a −80 °C freezer. The frozen tissue blocks were sectioned into 10 µm thickness using a cryostat microtome (CM1510‐S; Leica, Germany) and then immediately placed on histological glasses (Frontier FRC‐11; Matsunami, Japan) and dried. Tissue sections were made in triplicate per group, each from a different mouse. Once dried, OCT was removed from the tissue by submerging the slide glasses in PBS for 10 min. After the removal of OCT, OG488‐labelled 30Kc19α‐RUNX2 (1 µm) and OG488‐labelled HAB‐30Kc19α‐RUNX2 (1 µm) in PBS were applied to the tissue sections and incubated for 1 h at room temperature with gentle rocking. The slide glasses were rinsed three times with PBS to wash any unbound proteins and were coverslipped with the mounting solution. Binding affinities of OG488‐labelled 30Kc19α‐RUNX2 and OG488‐labelled HAB‐30Kc19α‐RUNX2 to various tissues were visualized by EVOS® FL auto cell imaging microscope (AMC1000; Thermo Fisher Scientific, USA). In the microscope, the bright field filter and green fluorescence filter were used, and the two images were merged using Image J software.

### In Vivo Protein Biodistribution

IVIS® Spectrum in vivo imaging (Perkin Elmer, USA) was taken to evaluate the binding affinity of HAB‐30Kc19α‐RUNX2 to the bone and to track the protein distribution in an animal. Before IVIS imaging, mice were fed with alfalfa free diet (AIN‐76A Rodent diet; Research diets, USA) for 5 days to prevent autofluorescence induced by diets. Balb/c‐nu female mice were divided into three groups (n = 3): mice administered with PBS, Atto550‐labelled 30Kc19α‐RUNX2, and Atto550‐labelled HAB‐30Kc19α‐RUNX2. PBS, Atto550‐labelled 30Kc19α‐RUNX2 (50 µg, 0.25 µg µL^−1^), and Atto550‐labelled HAB‐30Kc19α‐RUNX2 (50 µg, 0.25 µg µL^−1^) were injected via tail vein. After 2 h of injection, mice were sacrificed and major organs including the liver, heart, bone of femur and tibia, spleen, kidney, and lung were collected for fluorescence imaging. Fluorescence induced by protein distribution was visualized by IVIS® Spectrum in vivo imaging system using excitation 554 nm and emission 576 nm filter. For accurate comparison between different groups, the minimum and maximum values of all groups were unified (Min = 6.59e6, Max = 1.39e7). After fluorescence imaging, the average radiant efficiency of the region of interest (ROI) was measured using the corresponding software.

### Alkaline Phosphatase and Alizarin Red S Staining

Alkaline phosphatase (ALP) staining was conducted on recombinant protein‐treated hMSCs differentiated in OM for (7 and 14) days, and Alizarin Red S (ARS) staining for 21 days, to evaluate ALP activity and calcium deposition, respectively. Both staining were performed as described in the previous study.^[^ ^5^ ^]^ ARS quantification was performed by extracting cell‐bound ARS with 10% w/v cetylpyridinium chloride in deionized distilled water (DDW).^[^ ^33^ ^]^ Absorbance at 562 nm was measured with a microplate reader (Tecan), and data were normalized to the control group (without protein treatment).

### Real‐Time qRT‐PCR

To quantify osteoblast‐related gene expression, a real‐time quantitative reverse transcription polymerase chain reaction (qRT‐PCR) was conducted. Total RNA was isolated from three groups of hMSCs cultured in OM for (7, 14, and 21) days using AccuPrep® universal RNA extraction kit (Bioneer, Korea). Then, cDNAs were synthesized from the isolated RNAs by SuperiorScript III reverse transcriptase (Enzynomics). *GAPDH* was used as a reference gene, and three osteoblast‐related genes expressed in different stages of osteogenic differentiation were selected for target genes: *RUNX2*, *secreted phosphoprotein 1* (*SPP1*), and *bone gamma‐carboxyglutamate protein* (*BGLAP*). Forward and reverse primer (Bionics, Korea), cDNA, RNase‐free water, and SensiFAST SYBR (Bioline, USA), were mixed. Then, real‐time qRT‐PCR was carried out by StepOne^™^ real‐time PCR systems (Applied Biosystems, USA). *Glyceraldehyde‐3‐phosphate dehydrogenase (GAPDH)* was used as endogenous control. The relative fold induction (RFI) was calculated by ∆∆C_T_ method. Primer sequences are listed in Table S1 (Supporting Information).

### Luciferase Reporter Assay

A 2472 bp BSP promoter reporter plasmid containing the native BSP promoter fragment was provided by Renny Franceschi (University of Michigan Medical School).^[^ ^34^ ^]^ NIH3T3 cells were seeded in 96‐well plate (Eppendorf, Germany) at 2500 cells cm^2^, grown for 12 h, and 2472 bp BSP promoter reporter plasmid was delivered into NIH3T3 cells using Lipofectamine® 2000 reagent (Invitrogen) according to the manufacturer's instructions. After 48 h after the transfections, cells were treated with proteins for 4 h and then luciferase expression in the cells was assayed using the ONE‐Glo Luciferase Assay System. Transfer the cell lysates to 96‐well black plate (SPL Life Science, Korea) after 5 min of reagent addition. After that, measure a luminescence with a microplate reader (SPARK 10 M; Tecan, Switzerland).

### In Vivo Postmenopausal Osteoporosis Model

Female, 8‐week‐old Balb/c mice were purchased from Orient Bio and housed in pathogen‐free rooms under 12: 12 h of light‐dark cycles. OVX was performed to establish a postmenopausal osteoporosis model by following the protocol from the previously published papers.^[^ ^35^ ^]^ Before commencing OVX, Zoletil 50 (Virbac, France) and Rompun inj (Bayer, Germany) anesthetic agents were administered via intraperitoneal injection to alleviate animal pain. OVX was conducted by making a surgical incision on the dorsal midline approximately halfway between the middle of the back and the base of the tail.^[^ ^36^ ^]^ To reach the ovaries on both sides, a small retroperitoneal incision was also made bilaterally on the erector spinae muscle beneath the skin. When the ovaries surrounded by fats were exposed, ovaries and part of the oviduct on both sides were removed and skin wounds were closed by suture. Four weeks after OVX, the mice were divided into the following groups (n = 3): normal group without OVX, PBS group with OVX, 30Kc19α‐RUNX2 group with OVX, and HAB‐30Kc19α‐RUNX2 group with OVX. OVX mice were intravenously injected every week with PBS or 30Kc19α‐RUNX2 (50 µg, 1 µg µL^−1^) or HAB‐30Kc19α‐RUNX2 (50 µg, 1 µg µL^−1^) for 4 weeks. 50 µg of protein that shows low cytotoxicity was used for the animal experiment. After 4 weeks of protein injection, mice were sacrificed with carbon dioxide and tissues were collected for further evaluation. The uterus was also harvested and weighed to make sure that the postmenopausal osteoporosis model was well established (Figure S2, Supporting Information).

### Micro‐CT Analysis

Collected femur specimens were fixed with 4% PFA and scanned by microtomography (micro‐CT; Skyscan 1172; Bruker, USA). The image was taken under 142 µA of source current, 70 kV of source voltage, and 718 ms of exposure time. The image was reconstructed into a 3D image to evaluate trabecular bone regeneration using CT_vol_ software (Bruker) and bone volume to total volume fraction (BV/TV) was calculated using CT_An_ software (Bruker).

### Histological Analysis

Femur and spleen samples were collected and fixed with 4% PFA. After fixation, the femur was placed in 20% EDTA buffer in distilled water at 4 °C for 2 weeks for decalcification. The femur and spleen were then sequentially immersed in distilled water, 50% ethyl alcohol (EtOH; Duksan, Korea), 70% EtOH, 90% EtOH, 100% EtOH, and xylene (Daejung, Korea) for dehydration, and then embedded in paraffin. After paraffin blocks were cut into 10 µm thickness, H&E and MTC were performed.^[^ ^37^ ^]^ For bone regeneration analysis, histological staining was visualized by Eclipsed Ti2 inverted microscopy.

### Ethical Statement

All animal procedures were performed in accordance with Guide for the Care and Use of Laboratory Animals by Seoul National University (Approval No. SNU‐200511‐7‐2). All participants were aware of and in consent with the guidelines provided by the abovementioned committee.

### Statistical Analysis

All data present mean ± standard deviation (SD). Statistically significant differences were analyzed by one‐way ANOVA followed by Tukey's post hoc test. *p* values less than 0.05 were recognized as statistically significant: *p < 0.05, **p < 0.01, ***p < 0.001.

## Conflict of Interest

The authors declare no conflict of interest.

## Author Contributions

S.K. and H.L. contributed equally to this work. S.K., H.L., S.H. L.K., T.H.P, N.S.H designed the experiment; S.K., H.L., S.H. L.K., J.H., and E.K. conducted experiments; S.K., H.L., and J. H. performed data analysis; S.K. and H.L. wrote the manuscript. S.K., H. L., T.H.P., and N.S.H. contributed to scientific discussion of the article.

## Supporting information

Supporting Information

## Data Availability

The data that support the findings of this study are available from the corresponding author upon reasonable request.
