# Supplementary material for: Bone‐Targeted Delivery of Cell‐Penetrating‐RUNX2 Fusion Protein in Osteoporosis Model
Source: Adv Sci (Weinh). 2023 Aug 13;10(28):2301570. doi: 10.1002/advs.202301570 (PMC10558633; doi:10.1002/advs.202301570)
Supplement: Supplementary file 1 — Supporting Information [file ADVS-10-2301570-s001.pdf]

ADVANCED SCIENCE
Open Access

Supporting Information

for *Adv. Sci.*, DOI 10.1002/advs.202301570

Bone-Targeted Delivery of Cell-Penetrating-RUNX2 Fusion Protein in Osteoporosis Model

Seoyeon Kim, Haein Lee, Jiyeon Hong, Seung Hyun L. Kim, Euntaek Kwon, Tai Hyun Park and Nathaniel S. Hwang**

Supporting Information

Bone Targeting Delivery of Cell-Penetrating Protein-RUNX2 Fusion Protein Using Hydroxyapatite Binding Tag in Osteoporosis Model

Seoyeon Kim[1, †], Haein Lee[1, †], Jiyeon Hong[1], Seung Hyun L. Kim[2], Euntaek Kwon[2], Tai Hyun Park[1,2,3,4] and Nathaniel S. Hwang[1,2,3*]*

[1]S. Kim, H. Lee, J. Hong, Prof. T. H. Park, Prof. N. S. Hwang
School of Chemical and Biological Engineering, Institute of Chemical Processes, Seoul National University, 1 Gwanak-ro, Gwanak-gu, Seoul 08826, Republic of Korea
E-mail: nshwang@snu.ac.kr (N. S. Hwang) and thpark@ewha.ac.kr (T. H. Park)

[2]S. H. L. Kim, E. Kwon, Prof. T. H. Park, Prof. N. S. Hwang
Interdisciplinary Program in Bioengineering, Seoul National University, 1 Gwanak-ro, Gwanak-gu, Seoul 08826, Republic of Korea

[3]Prof. T. H. Park, Prof. N. S. Hwang
BioMAX/N-Bio Institute, Institute of BioEngineerig, Seoul National University, 1 Gwanakro, Gwanak-gu, Seoul 08826, Republic of Korea

[4]Prof. T. H. Park
Department of Nutritional Science and Food Management, Ewha Womans University, 52, Ewhayeodae-gil, Seodaemun-gu, Seoul 03760, Republic of Korea

[†]S. Kim and H. Lee contributed equally to this work.

[*]To whom correspondence should be addressed.
School of Chemical and Biological Engineering, Institute of Chemical Processes, Seoul National University, Seoul 08826, Republic of Korea.
Email address: nshwang@snu.ac.kr (N. S. Hwang) and thpark@ewha.ac.kr (T. H. Park)

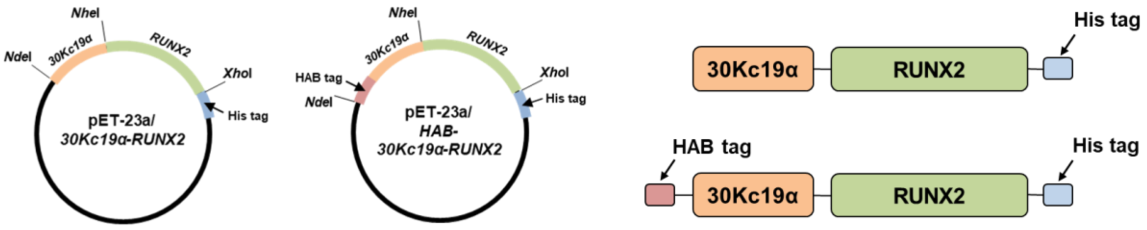

MDDDDDDDCADSDVPNDILEEQLYNSVVVADYDSAVEKSKHLYEEKKSEVITNVVNKLIRNNKMNCMEYAY
QLWLQGSKDIVRDCFPVEFRLIFAENASMASNSLFSAVTPCQQSFFWDPSTSRRFSPPSSSLQPGKMSDVSPV
VAAQQQQQQQQQQQQQQQQQQQQQQQQQQQQQEAAAAAAAAAAAAAAAAAVPRLRPPHDNRT
MVEIIADHPAELVRTDSPNFLCSVLPSHWRCNKTLPVAFKVVALGEVPDGTVVTVMAGNDENYSAELRNASA
VMKNQVARFNDLRFVGRSGRGKSFTLTITVFTNPPQVATYHRAIKVTVDGPREPRRHRQKLDDSKPSLFSDRL
SDLGRIPHPSMRVGVPPQNPRPSLNSAPSPFNPQGQSQITDPRQAQSSPPWSYDQSYPSYLSQMTSPSIHST
TPLSSTRGTGLPAITDVPRRISDDDTATSDFCLWPSSLSKKSQAGASELGPFSDPRQFPSISSLTESRFSNPRMHY
PATFTYTPPVTSGMSLGMSATTHYHTYLPPPYPGSSQSQSGPFQTSSTPYLYYGTSSASYQFPMVPGGDRSPS
RMVPPCTTTSNGSTLLNPNLPNQNDGVDADGSHSSSPTVLNSSGRMDESVWRPYPKKKRKVLEHHHHHH

Figure S1. Amino acid sequences of HAB-30Kc19α-RUNX2. The amino acid sequences of 30Kc19α and RUNX2 are indicated by orange and green letters, respectively. HAB tag (DDDDDDDC) and His tag are located at the N-terminus and C-terminus of recombinant proteins.

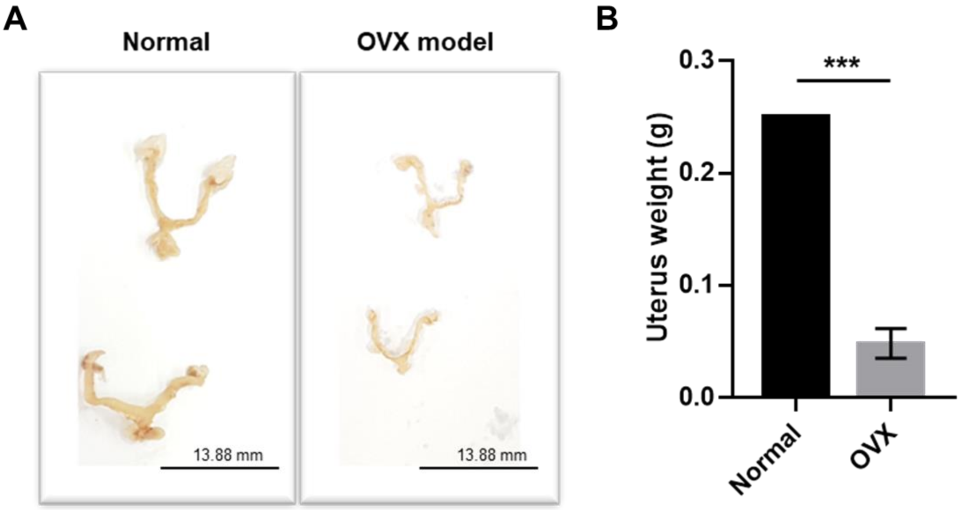

Figure S2. Establishment of a mouse model for osteoporosis. The osteoporosis model was generated by the removal of ovaries. (A) Morphological changes in the uterus after ovariectomy (OVX). The uterus size of the OVX became smaller than that of the normal group. Scale bar: 13.88 mm (B) Weight changes in the uterus after OVX. Reduction in the uterus weight confirming successful ovary removal. ***$p < 0.001$

Gene	Primer sequence (5'→3')	
GAPDH	**Forward**	TTCACCACCATGGAGAAGGC
	**Reverse**	TCTTCTGGGTGGCAGTGATG
RUNX2	**Forward**	GCGCATTCCTCATCCCAGTA
	**Reverse**	GGCTCAGGTAGGAGGGGTAA
SPP1	**Forward**	GCATCACCTGTGCCATACCAG
	**Reverse**	TCCTGGCTGTCCACATGGTC
BGLAP	**Forward**	TATTGGCCCTGGCCGCACTT
	**Reverse**	CTCACACACCTCCCTCCTG

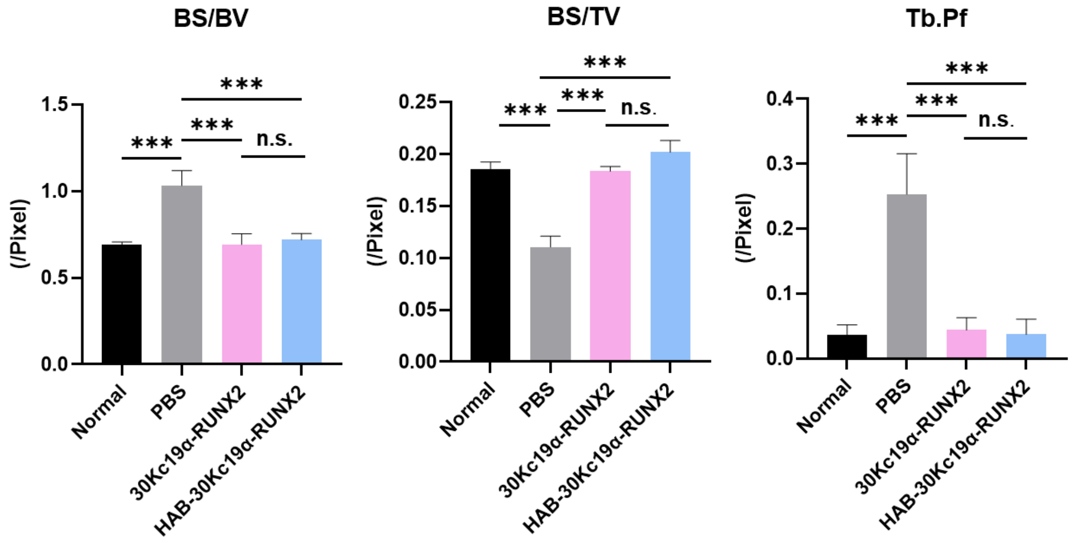

Figure S3. In vivo bone regeneration of recombinant protein treatment in postmenopausal osteoporosis model. Four weeks after the osteoporosis model was generated by ovariectomy (OVX) in Balb/c mice, PBS (50 µL), 30Kc19α-RUNX2 (50 µg, 1 µg μL^{-1}) and HAB-30Kc19α-RUNX2 (50 µg, 1 µg μL^{-1}) were injected via tail vein injection four times every 1 week (n=3). Micro-CT parameters such as bone surface to bone ratio (BS/BV), bone surface density (BS/TV), trabecular pattern factor (Tb.Pf) was measured. ***$p < 0.001$

Table S1. Primer sequences used in qRT-PCR.
